# Virulence genes and antimicrobial susceptibility of lactose-negative and lactose-positive strains of *Escherichia coli* isolated from pregnant women and neonates

**DOI:** 10.1007/s12223-017-0506-y

**Published:** 2017-02-24

**Authors:** Agnieszka Kaczmarek, Krzysztof Skowron, Anna Budzyńska, Katarzyna Grudlewska, Eugenia Gospodarek-Komkowska

**Affiliations:** Department of Microbiology, Nicolaus Copernicus University in Torun, Ludwik Rydygier Collegium Medicum, 9 M. Skłodowskiej-Curie Street, 85-094 Bydgoszcz, Poland

## Abstract

*Escherichia coli* can cause serious infections in the neonates and pregnant women. Although *E. coli* is widely studied, *E. coli* lactose-negative (lac−) strains have been rarely described before. So, the aim of this study was to compare lac− and lactose-positive (lac+) *E. coli* strains in respect of antimicrobial susceptibility and the frequency of virulence genes (VGs). The study included 58 lac+ and 58 lac− *E. coli* strains isolated from pregnant women and neonates. Culture and the results of biochemical reactions were conducted for lac− and lac+ *E. coli* identification and differentiation. Disc diffusion test was performed to study the antimicrobial susceptibility of the isolates, and PCR was used to detect VGs. Resistance to at least one of the tested antibiotics was found among 14 (25.9%) *E. coli* lac+ and in 26 (44.9%) *E. coli* lac− strains. Both lac+ and lac− *E. coli* strains were mostly resistant to ampicillin (22.4 and 39.7%) and ticarcillin (20.7 and 39.7%). None of the tested strains produced extended-spectrum β-lactamases (ESBLs). Genes *fimH*, *fimA*, *iutA*, *sfa/foc*, *neuC*, *ibeA*, and *hlyF* were detected, respectively, in 96.6, 82.8, 32.8, 24.1, 22.4, 12.1, and 6.9% of lac+ *E. coli* strains and in 94.8, 86.2, 48.3, 19.0, 8.6, 8.6, and 1.7% of lac− strains. The antimicrobial susceptibility and the pathogenic potential of both tested groups of *E. coli* strains are similar. Therefore, omitting *E. coli* lac− strains as a potential etiological agent of infections may pose a threat to the health and life of both mothers and neonates.

## Introduction

Saprophytic *Escherichia coli* strains, colonizing the human alimentary tract, perform the function of commensals. In the case when those bacteria colonize the human organism beyond the alimentary tract, they become opportunistic pathogens (Tenaillon et al. 2010). Most often, *E. coli* strains can cause urinary tract infections (UTIs) and intestinal infections, both in children and in adults. In rarer cases, they are also responsible for generalized infections such as bacteremia, sepsis, and meningitis in neonates (Korczak et al. 2005; Obata-Yasuoka et al. 2002; Watt et al. 2003). An infection of a neonate may occur during the birth, when the child is exposed to a direct contact with bacteria present in the genital tract and sometimes also with microorganisms derived from the gastrointestinal tract of the woman giving birth (Watt et al. 2003). Colonization of the genital tract or anus of a pregnant woman by *E. coli* strains may also lead to infection of a neonate by ascending route. This infection happens most frequently as a result of premature rupture of membranes. Microorganisms from the lower part of the genital tract (vagina, uterine cervix) get to the amniotic sac and infect the fetus (Newton 2005). Also, UTIs occurring in pregnant women are serious threat to the fetus (Barber et al. 2013). Asymptomatic bacteriuria is the most often type of UTIs in pregnant women which, if not treated, in 20.0–30.0% of cases may lead to pyelonephritis. It may result in premature birth and developmental retardation or fetus necrosis, as well as a fetus with low birth weight (Janicka et al. 1997). Development of an infection largely depends on immunity of the host organism and the pathogenicity of the strain that causes it (Korczak et al. 2005). Adhesins, capsular antigen K1, hemolysins, aerobactin, and invasion protein IbeA play the essential role in the pathogenesis of infections caused by *E. coli* (Johnson 1991)*.* Most *E. coli* rods are biochemically active. Their most important traits are the ability to decompose tryptophan into indole and to ferment lactose. However, in clinical material, the presence of non-reactive *E. coli* strains may be indicated. Their identification is difficult due to low metabolic activity, including the lack of the ability to lactose fermentation (Gadage et al. 2014). Lactose-negative (lac−) strains of *E. coli*, just as lactose-positive (lac+) strains, can have a number of virulence factors (VFs) and exhibit antibiotic resistance, as well as induce infections (Nicoletti et al. 1988). In the available references, there is little information about *E. coli* lac− strains. There are also no results of studies comparing these strains with *E. coli* lac+. Accordingly, the aim of this study was to investigate *E. coli* lac− strains isolated from pregnant women and neonates in respect of antibiotic susceptibility, the ability to produce the extended-spectrum β-lactamases (ESBLs), and the frequency of genes encoding selected VFs. The obtained data were compared with results obtained for *E. coli* lac+ strains.

## Material and methods

### Bacterial strains and its identification

The research material enclosed 116 genetically unrelated *E. coli* strains (58 lac+ and 58 lac−) isolated from pregnant women and neonates hospitalized at the Dr. Biziel University Hospital No.2 in Bydgoszcz. *E. coli* lac+ strains from pregnant women were obtained from rectal swabs (*n* = 26), vaginal swabs (*n* = 12), and urine (*n* = 8) and, in the case of neonates, from nasal cavities (*n* = 10) and urine (*n* = 2). In turn, *E. coli* lac− strains were isolated from rectal swabs (*n* = 52) and urine (*n* = 1) of pregnant women and from nasal cavities (*n* = 4) and urine (*n* = 1) of neonates.

*E. coli* species identification was based upon colony morphology, the ability to ferment lactose on the MacConkey agar (Becton Dickinson), and the results of biochemical reactions obtained in the VITEK®2 Compact (bioMérieux). *E. coli* lac+ and lac− strains were differentiated on the basis of colony color on the MacConkey agar and the result of the Taxo ONPG Disks test (Becton Dickinson).

### Antimicrobial susceptibility testing and detection of ESBLs

The susceptibility of *E. coli* strains to selected antibiotics was assessed with the Kirby-Bauer disk-diffusion method on the Mueller-Hinton agar (Becton Dickinson) according to the recommendations of the European Committee on Antimicrobial Susceptibility Testing (EUCAST 2013). *E. coli* ATCC 25922 was used for quality-control purposes.

The production of ESBLs was assessed using the double-disk synergy test (Jarlier et al. 1988). *E. coli* ATCC 25922 (not producing ESBLs) and *Klebsiella pneumoniae* ATCC 700603 (producing ESBLs) were used as reference strains.

### Detection of virulence genes

In order to detect VGs, isolation of DNA and multiplex PCR reactions were performed. DNA from the tested *E. coli* strains was isolated using the Genomic Mini kit (A&A Biotechnology). The presence of genes encoding the following VFs: type 1 fimbriae (*fimA* and *fimH*), S and F1C fimbriae (*sfa/foc*), antigen K1 (*neuC*), receptor for aerobactin (*iutA*), and protein IbeA (*ibeA*), hemolysin HlyF (*hlyF*), was detected with multiplex PCR, according to the authors’ methodology described before by Kaczmarek et al. (2012). The *E. coli* BEN2908 strain was used as positive control for the *fimA*, *fimH*, *neuC*, *hlyF*, *iutA*, and *ibeA* genes and strain 536 for *sfa/foc*. As a negative control, a reaction mixture without DNA was included in the experiment.

### Statistical analysis

Statistical analysis of the results was carried out using the chi-square test and Fisher exact test, at the significance level α = 0.05. Calculations were made with the program Statistica 10 PL (StatSoft, Poland).

## Results

### Antibiotic susceptibility analysis

It was indicated that *E. coli* strains, both lac+ and lac−, were mostly resistant to ampicillin (22.4 and 39.7%) and ticarcillin (20.7 and 39.7%). Moreover, in the group of *E. coli* lac− strains, a high percentage of strains not susceptible to ticarcillin with clavulanic acid (19.0%) and cotrimoxazole (10.3%) was determined. From 1.7 to 6.9% of *E. coli* lac+ and lac− strains were resistant to the other tested antibiotics (Table 1). The statistically significant differences between *E. coli* lac+ and lac− were found in the resistance to ticarcillin (*P* < 0.05) and ticarcillin with clavulanic acid (*P* < 0.05). A significantly higher percentage of strains resistant to these antibiotics was recorded in the group of *E. coli* lac− strains.

**Table 1:** Comparison of the antimicrobial susceptibility between *E. coli* lac+ (*n* = 58) and *E. coli* lac− strains (*n* = 58)

Antibiotic	No. of *E. coli* lac+			No. of *E. coli* lac−		
	R (%)	I (%)	S (%)	R (%)	I (%)	S (%)
AM	13 (22.4)	0 (0.0)	45 (77.6)	23 (39.7)	0 (0.0)	35 (60.3)
SAM	0 (0.0)	0 (0.0)	58 (100.0)	4 (6.9)	0 (0.0)	54 (93.1)
AMC	0 (0.0)	0 (0.0)	58 (100.0)	2 (3.4)	0 (0.0)	56 (96.6)
TIC	12 (20.7)	0 (0.0)	46 (79.3)	23 (39.7)	0 (0.0)	35 (60.3)
TIM	0 (0.0)	0 (0.0)	58 (100.0)	11 (19.0)	0 (0.0)	47 (81.0)
FOX	1 (1.7)	0 (0.0)	57 (98.3)	0 (0.0)	0 (0.0)	58 (100.0)
CXM	0 (0.0)	0 (0.0)	58 (100.0)	0 (0.0)	0 (0.0)	58 (100.0)
FEP	0 (0.0)	0 (0.0)	58 (100.0)	0 (0.0)	0 (0.0)	58 (100.0)
IPM	0 (0.0)	0 (0.0)	58 (100.0)	0 (0.0)	0 (0.0)	58 (100.0)
ATM	0 (0.0)	3 (5.2)	55 (94.8)	0 (0.0)	0 (0.0)	58 (100.0)
CIP	0 (0.0)	3 (5.2)	55 (94.8)	2 (3.4)	0 (0.0)	56 (96.6)
NOR	0 (0.0)	3 (5.2)	55 (94.8)	2 (3.4)	1 (1.7)	55 (94.8)
AN	0 (0.0)	0 (0.0)	58 (100.0)	0 (0.0)	0 (0.0)	58 (100.0)
GM	0 (0.0)	0 (0.0)	58 (100.0)	1 (1.7)	0 (0.0)	57 (98.3)
NN	0 (0.0)	1 (1.7)	57 (98.3)	0 (0.0)	1 (1.7)	57 (98.3)
TGC	0 (0.0)	0 (0.0)	58 (100.0)	0 (0.0)	0 (0.0)	58 (100.0)
C	1 (1.7)	0 (0.0)	57 (98.3)	2 (3.4)	0 (0.0)	56 (96.6)
SXT	3 (5.2)	0 (0.0)	55 (94.8)	6 (10.3)	0 (0.0)	52 (89.7)

Underlined value are differences remarkable although not statistically significant

*R* resistant, *I* intermediate, *S* susceptible, *AM* ampicillin, *SAM* ampicillin/sulbactam, *AMC* amoxicillin/clavulanic acid, *TIC* ticarcillin, *TIM* ticarcillin/clavulanic acid, *FOX* cefoxitin, *CXM* cefuroxime, *FEP* cefepime, *IPM* imipenem, *ATM* aztreonam, *CIP* ciprofloxacin, *NOR* norfloxacin, *AN* amikacin, *GM* gentamicin, *NN* tobramycin, *TGC* tigecycline, *C* chloramphenicol, *SXT* trimethoprim/sulfamethoxazole

None of the tested *E. coli* lac+ and lac− strains produced ESBL.

Among the tested *E. coli* strains, 20 antimicrobial resistance patterns were distinguished (Table 2). It was stated that 4 profiles (A–D) were common for both the tested groups of *E. coli*, whereas 7 (E–K) were typical only for *E. coli* lac+ strains and 9 (L–U) of *E. coli* lac−. Profile A occurred statistically significantly more frequently in *E. coli* lac+ (*n* = 43) than in *E. coli* lac− (*n* = 32) (*P* < 0.05) (Table 2). Among profiles typical only for *E. coli* lac+, two strains belonged to profile E and the other profiles (F–K) were represented by single *E. coli* strains. However, among profiles determined only for *E. coli* lac−, most isolates (*n* = 6) were classified to profile L, in which a statistically significant difference was indicated between *E. coli* lac+ and *E. coli* lac− strains (*P* < 0.05) (Table 2).

**Table 2:** Antimicrobial resistance patterns in *E. coli* lac+ (*n* = 58) and *E. coli* lac− (*n* = 58) strains

Profile	Antibiotic(s)	Total no. of *E. coli* (%)	No. of *E. coli* lac+ (%)	No. of *E. coli* lac− (%)	*P* value
A	R^*^: --- I^**^: ---	75 (64.7)	**43 (37.1)**	**32 (27.6)**	**0.019**
B	R: AM, TIC	12 (10.3)	5 (4.3)	7 (6.0)	0.542
C	R: AM, TIC, SXT	4 (3.4)	1 (0.9)	3 (2.6)	0.309
D	R: C	2 (1.7)	1 (0.9)	1 (0.9)	1.000
E	R: AM, TIC I: CIP	2 (1.7)	2 (1.7)	0 (0.0)	0.154
F	R: AM, TIC,SXT I:CIP, NOR	1 (0.9)	1 (0.9)	0 (0.0)	0.315
G	R: AM, TIC, SXT I: NOR	1 (0.9)	1 (0.9)	0 (0.0)	0.315
H	R: AM, TIC I: ATM, NOR	1 (0.9)	1 (0.9)	0 (0.0)	0.315
I	R: AM, TIC, I: ATM	1 (0.9)	1 (0.9)	0 (0.0)	0.315
J	R: AM, FOX	1 (0.9)	1 (0.9)	0 (0.0)	0.315
K	I: ATM, NN	1 (0.9)	1 (0.9)	0 (0.0)	0.315
L	R: AM, TIC, TIM	6 (5.2)	**0 (0.0)**	**6 (5.2)**	**0.012**
M	R: AM, SAM, AMC, TIC, TIM	2 (1.7)	0 (0.0)	2 (1.7)	0.154
N	R: AM, TIC, TIM, CIP, NOR, C, SXT	1 (0.9)	0 (0.0)	1 (0.9)	0.315
O	R: AM, TIC, GM, SXT I: NN	1 (0.9)	0 (0.0)	1 (0.9)	0.315
P	R: AM, TIC, TIM I: NOR	1 (0.9)	0 (0.0)	1 (0.9)	0.315
R	R: AM, SAM, TIC, TIM	1 (0.9)	0 (0.0)	1 (0.9)	0.315
S	R: AM, SAM, TIC	1 (0.9)	0 (0.0)	1 (0.9)	0.315
T	R: CIP, NOR	1 (0.9)	0 (0.0)	1 (0.9)	0.315
U	R: SXT	1 (0.9)	0 (0.0)	1 (0.9)	0.315
	Total	116 (100.0)	58 (50.0)	58 (50.0)	

Bold values are differences statistically significant. Underlined value are differences remarkable although not statistically significant

*R* resistant, *I* intermediate, *AM* ampicillin, *SAM* ampicillin/sulbactam, *AMC* amoxicillin/clavulanic acid, *TIC* ticarcillin, *TIM* ticarcillin/clavulanic acid, *FOX* cefoxitin, *CXM* cefuroxime, *FEP* cefepime, *IPM* imipenem, *ATM* aztreonam, *CIP* ciprofloxacin, *NOR* norfloxacin, *AN* amikacin, *GM* gentamicin, *NN* tobramycin, *TGC* tigecycline, *C* chloramphenicol, *SXT* trimethoprim/sulfamethoxazole

Moreover, 8 out of 20 drug-susceptibility profiles were represented also by *E. coli* strains intermediate to 1 (profiles E, G, I, O, P) and 2 antibiotics (profiles F, H, K). A higher percentage of antibiotic intermediate-susceptible strains was observed among *E. coli* lac+ strains than in the case of *E. coli* lac− strains (12.1 vs. 3.4%). One isolate of *E. coli* lac+ showed only an intermediate susceptibility to two tested antibiotics, at the same time not being resistant to any of the tested drugs (profile K) (Table 2).

### Prevalence of VGs

**Fig. 1:**
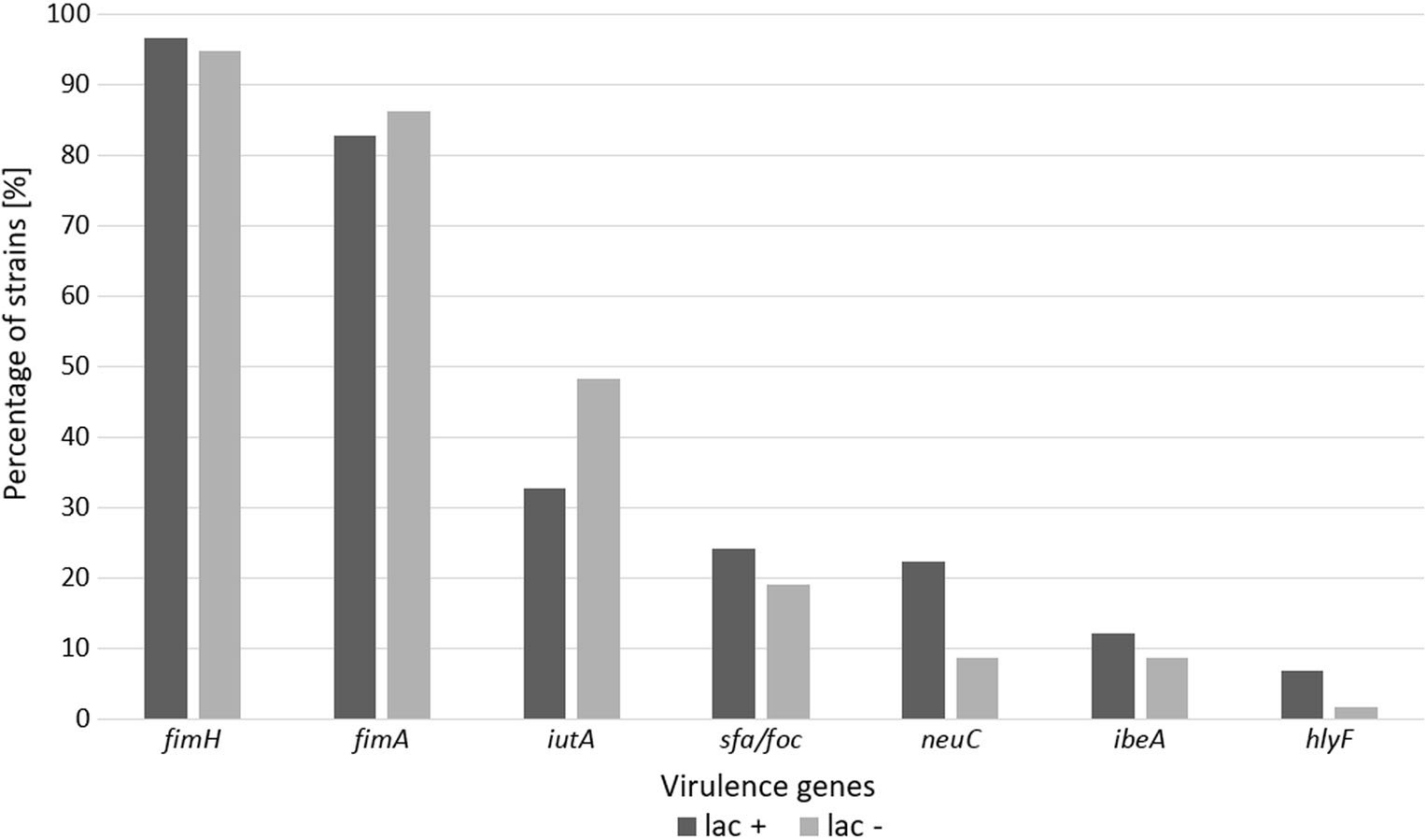
Prevalence of VGs in *E. coli* lac+ and *E. coli* lac− strains

It was stated that the most common virulence genes (VGs), in *E. coli* lac+ and lac− strains, were *fimH* (96.6 and 94.8%, respectively) and *fimA* (82.8 and 86.2%, respectively) (Fig. 1). A considerable percentage of *E. coli* strains (32.8% lac+ and 48.3% lac−) had the gene *iutA.* On the other hand, the presence of genes *sfa*/*foc*, *neuC*, and *ibeA* was found, respectively, in 24.1, 22.4, and 12.1% of *E. coli* lac+ rods and in 19.0, 8.6, and 8.6% of lac−. The gene *hlyF* occurred the least commonly among the tested isolates (6.9% of lac+ and 1.7% of lac−). A statistically significant difference between the compared groups of *E. coli* lac+ and lac− strains was observed only in the case of the gene *neuC* (*P* < 0.05) (Fig. 1).

This study indicated that *E. coli* strains represented different profiles of genes encoding VFs. In the tested populations, 20 profiles of VGs were identified, including 9 common for *E. coli* lac+ and *E. coli* lac− strains as well as 8 typical only for *E. coli* lac+ and 3 of *E. coli* lac− (Table 3). Profile II occurred statistically significantly more frequently in *E. coli* lac− (*n* = 16) than in *E. coli* lac+ (*n* = 5) (*P* < 0.05) (Table 3).

**Table 3:** Profiles of VGs in *E. coli* lac+ (*n* = 58) and *E. coli* lac− (*n* = 58) strains

	Gene(s)	Total no. of *E. coli* (%)	No. of *E. coli* lac+ (%)	No. of *E. coli* lac−(%)	*P* value
I	fimA, fimH	44 (37.9)	24 (20.7)	20 (17.2)	0.444
II	fimA, fimH, iutA	21 (18.1)	**5 (4.3)**	**16 (13.8)**	**0.008**
III	sfa/foc, fimA, fimH, iutA	9 (7.8)	3 (2.6)	6 (5.2)	0.298
IV	fimH	8 (6.9)	4 (3.4)	4 (3.4)	1.000
V	sfa/foc, fimA, fimH	5 (4.3)	3 (2.6)	2 (1.7)	0.648
VI	neuC, sfa/foc, fimA, fimH, ibeA	4 (3.4)	1 (0.9)	3 (2.6)	0.309
VII	neuC, fimA, fimH, iutA	4 (3.4)	3 (2.6)	1 (0.9)	0.309
VIII	neuC, fimH, iutA	3(2.6)	2 (1.7)	1 (0.9)	0.559
IX	Lack of tested genes	3 (2.6)	2 (1.7)	1(0.9)	0.559
X	neuC, sfa/foc, fimA, fimH, hlyF, iutA, ibeA	2 (1.7)	2 (1.7)	0 (0.0)	0.154
XI	neuC, sfa/foc, fimA, fimH, ibeA, iutA	2 (1.7)	2 (1.7)	0 (0.0)	0.154
XII	neuC, sfa/foc, fimA, fimH	2 (1.7)	2 (1.7)	0 (0.0)	0.154
XIII	sfa/foc, fimA, fimH, ibeA	1 (0.9)	1 (0.9)	0 (0.0)	0.315
XIV	fimA, fimH, hlyF, iutA	1 (0.9)	1 (0.9)	0 (0.0)	0.315
XV	neuC, fimA, fimH	1 (0.9)	1 (0.9)	0 (0.0)	0.315
XVI	fimH, hlyF, ibeA	1 (0.9)	1 (0.9)	0 (0.0)	0.315
XVII	fimH, iutA	1 (0.9)	1 (0.9)	0 (0.0)	0.315
XVIII	fimA, fimH, iutA, ibeA	2 (1.7)	0 (0.0)	2 (1.7)	0.154
XIX	hlyF, iutA	1 (0.9)	0 (0.0)	1 (0.9)	0.315
XX	iutA	1 (0.9)	0 (0.0)	1 (0.9)	0.315
Total		116 (100.0)	58 (50.0)	58 (50.0)	

Bold values are differences statistically significant. Underlined values are differences remarkable although not statistically significant

**Fig. 2:**
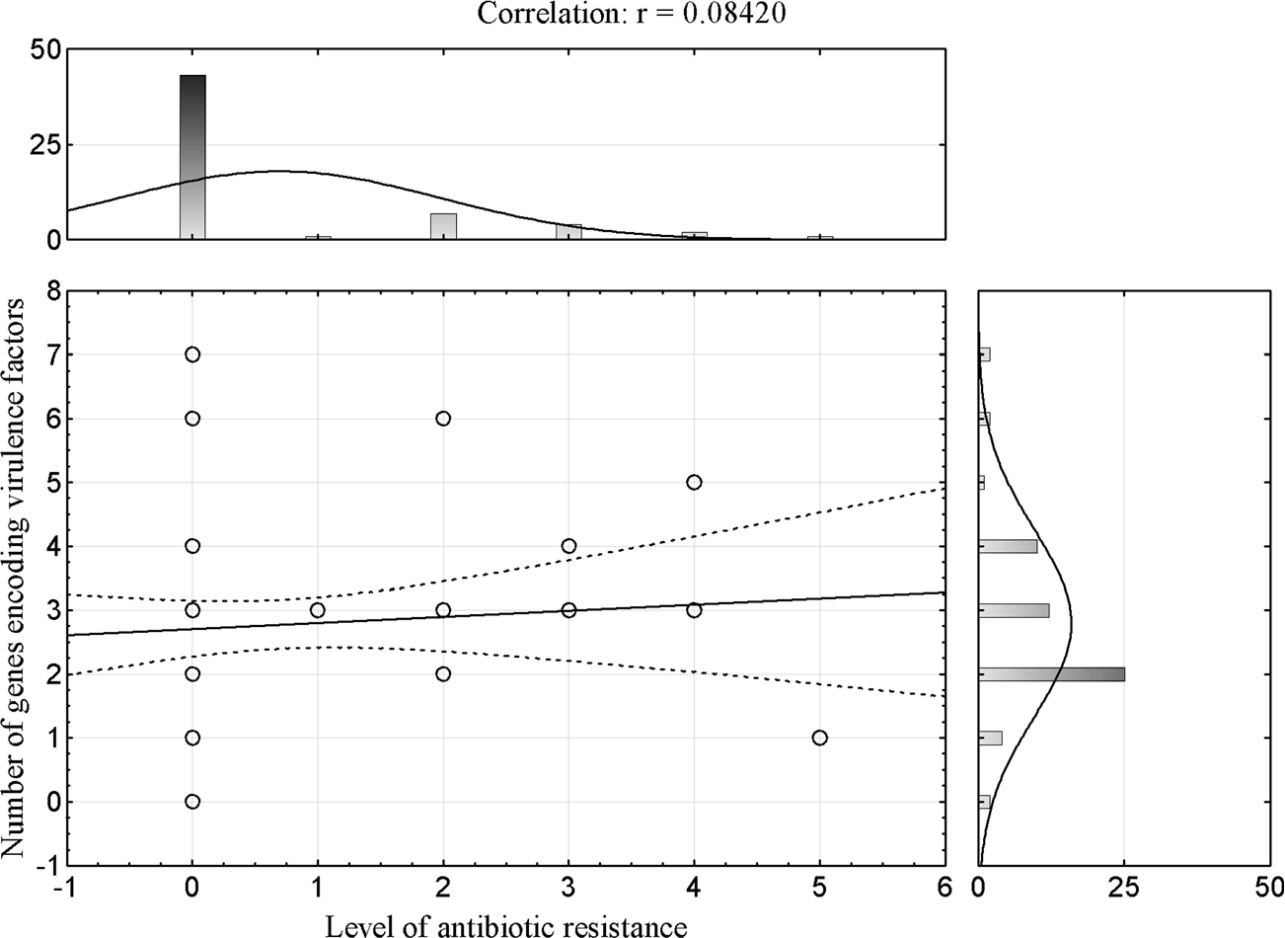
Correlation between antibiotic resistance and number of tested VGs for *E. coli* lac+

**Fig. 3:**
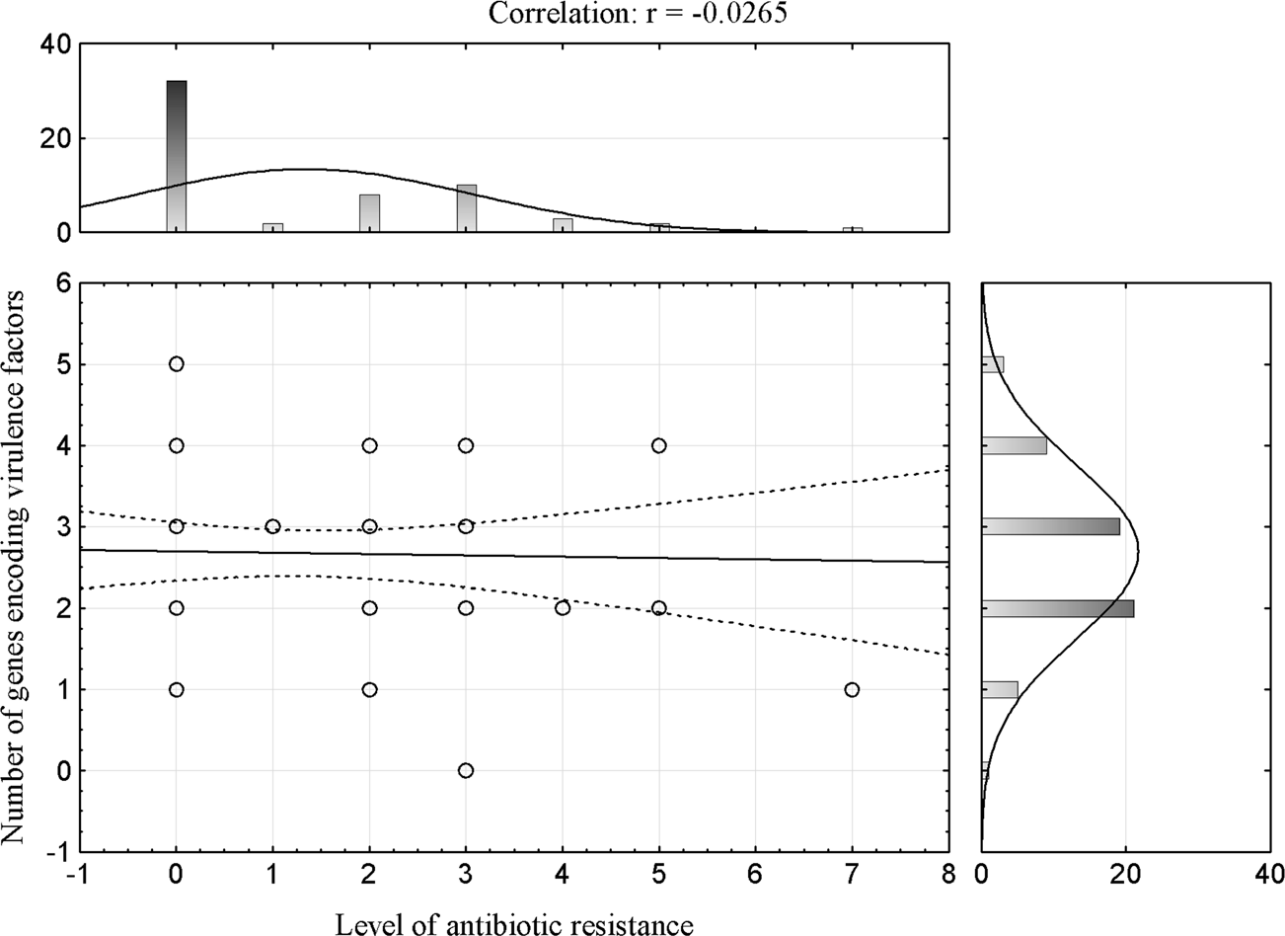
Correlation between antibiotic resistance and number of tested VGs for *E. coli* lac−

For *E. coli* lac+ strains, a statistically insignificant (*P* > 0.05) but slightly positive correlation (*r* = 0.08) between the number of antibiotics to which the tested strains were resistant and the number of VGs which they had was showed (Fig. 2). From this, it follows that there was a very weakly drawn tendency to increase the virulence of strains along with an increase in their antibiotic resistance. In contrast, for *E. coli* lac− strains, a statistically insignificant (*P* > 0.05) but slightly negative correlation (*r* = −0.03) was shown (Fig. 3).

## Discussion

*E. coli* should be ranked into the most important bacterial agents that can cause life-threatening infections in the perinatal period (Watt et al. 2003). These bacteria may be the cause of meningitis in neonates (neonatal meningitis-associated *E. coli*, NEMEC), bacteriemia and sepsis (sepsis-associated *E. coli*, SEPEC), and urinary tract infections (uropathogenic *E. coli*, UPEC) (Obata-Yasuoka et al. 2002; Watt et al. 2003; Alemu et al. 2012).

The analysis of the drug-susceptibility profiles of pathogenic bacterial strains enables the constant monitoring of their resistance to antibiotics. However, difficulty results from the fact that the authors most often do not report to which group (lac+ or lac−) they classified the tested *E. coli* strains. Therefore, when discussing the obtained present results, it is impossible to state to which population of *E. coli* we refer.

Susceptibility of *E. coli* strains to semi-synthetic penicillins is diverse in the world. In Poland (Janicka et al. 1997), Nigeria (Poey et al. 2012), and Ethiopia (Alemu et al. 2012; Tadesse et al. 2014), it was estimated that 80.8, 95.3, and 68.8–100% of *E. coli* strains were resistant to ampicillin, respectively. A lower percentage of *E. coli* strains resistant to ampicillin isolated from the vagina and rectum of pregnant women (39.9%), as well as those not pregnant (48.6%), was found by Hilbert et al. (2009) and Villar et al. (2013) and in the present study (31.0%). Moreover, in this study, a statistically significant difference was indicated between *E. coli* lac+ and lac− strains in resistance to ticarcillin (20.7 vs 39.7%). Also, a low percentage of *E. coli* strains resistant to penicillins with inhibitors of beta-lactamases and cephalosporins were found in this study. Significant differences between *E. coli* lac+ and lac− strains were found only in the case of resistance to ticarcillin with clavulanic acid (0.0 vs 19.0%). Similar results were also obtained in the authors’ earlier study (Kaczmarek et al. 2011), as well as in studies by other authors (Barcaite et al. 2012), irrespective of the fact if the strains were isolated from the rectum, vagina, or urine of pregnant women.

In the presented study, all the *E. coli* lac+ and lac− strains were susceptible to imipenem, amikacin, and tigecycline, which was also indicated in several other centers in Poland (Daniluk et al. 2008; Cisowska et al. 2001; Sobieszczańska et al. 2003; Sękowska et al. 2009). All the tested *E. coli* lac− strains and 94.8% of lac+ strains were also susceptible to aztreonam. Similar results were recorded in the earlier studies in Poland (Kaczmarek et al. 2011; Cisowska et al. 2001).

Moreover, a high percentage of *E. coli* strains from both tested groups susceptible to ciprofloxacin, norfloxacin, gentamicin, tobramycin, chloramphenicol, and trimethoprim/sulfamethoxazole was observed in the present study. An equally high percentage of strains susceptible to ciprofloxacin, with no resistance to gentamicin and amikacin, was isolated from neonates in Lithuania (98.3%) (Tamelienė et al. 2012). A high percentage of strains susceptible to ciproflofloxacin and aminoglycosides was also recorded in *E. coli* isolated from the vagina and rectum of pregnant women (Barcaite et al. 2012). Mehta et al. (2012) and Enayat et al. (2008) recorded a lower percentage of *E. coli* strains susceptible to fluoroquinolones and trimethoprim/sulfamethoxazole, respectively. Other authors that used *E. coli* isolated from the vagina of pregnant women reported almost 92% of the strains susceptible to cotrimoxazole (Spaetgens et al. 2002).

ESBLs producing *E. coli* strains are a serious therapeutic and epidemiological problem (Lina et al. 2014). In this study, none of the tested strains produced ESBLs. In the earlier study, also, no strains producing those enzymes were detected in *E. coli* isolated from pregnant women and neonates (Kaczmarek et al. 2011). In contrast, Villar et al. (2013) found the ability to produce ESBL enzymes in 5.4% of *E. coli* strains isolated from pregnant women.

The knowledge about etiological agents of an infection and their resistance to drugs, as well as the identification of traits responsible for the virulence of bacteria, is of the utmost importance in predicting the clinical course of an infection. The information about the virulence of the *E. coli* strain also facilitates making proper therapeutic decision.

In this study, in both groups of *E. coli* rods, more than 80% of strains having the gene *fimA* encoding the major structural subunit of type 1 fimbriae were identified. A similar result was obtained in the authors’ earlier study (Kaczmarek et al. 2012), as well as in the study by Micenková et al. (2014). On the other hand, the presence of the gene *fimH* determining the biosynthesis of the adhesin of those fimbriae was found in more than 90% of all the tested *E. coli* strains in the present study. Similarly, other authors indicated the presence of this gene among 90–100% of strains collected both from pregnant (Obata-Yasuoka et al. 2002; Poey et al. 2012; Al-Mayahie 2013) and not pregnant women (Obata-Yasuoka et al. 2002; Al-Mayahie 2013).

S- and F1C/S-related fimbriae are encoding by the genes *sfa* and *foc*, respectively (Juskova and Ciznar 1994). Watt et al. (2003) and Al-Mayahie (2013) indicated the presence of genes encoding S and F1C fimbriae in 48–60% of strains isolated from the vagina of pregnant women. In this study, the frequency of the gene *sfa/foc* was lower and amounted to 24.1% among *E. coli* lac+ strains and 19.0% among lac− strains.

In the presented study, the occurrence of the gene *hlyF*, encoding hemolysin F, was found in only 4.3% of strains, and it occurred more often in lac+ strains. In an earlier study, the percentage of strains that had the gene *hlyF* was significantly higher in *E. coli* K1 (28.4%), in comparison with the group of bacteria without this antigen (6.0%) (Kaczmarek et al. 2012).

*E. coli* producing the K1 antigen are responsible for about 80% of cases of meningitis in neonates (Watt et al. 2003). Moreover, they may be the cause of bacteremia, sepsis, and UTIs, both in children and adults (Alemu et al. 2012). The presence of the gene *neuC*, encoding the capsule K1, was higher in strains isolated from neonates with meningitidis (92%) (Ewers et al. 2007; Watt et al. 2003) than in strains isolated from the vagina, rectum, or urine of pregnant women (Watt et al. 2003). In the present study, *E. coli* lac+ strains had significantly more often gene *neuC* (22.4%) in comparison with the group of lac− strains (8.6%).

This study indicated the presence of the gene *iutA*, encoding the aerobactin receptor, in more than 30% of the tested *E. coli* lac+ strains and in almost 50% of *E. coli* lac− strains. A similar result was obtained by Poey et al. (2012) and Watt et al. (2003). In the study by Obata-Yasuoka et al. (2002), there were more often isolated strains having the gene-coding aerobactin from non-pregnant women than from pregnant ones.

Invasive protein IbeA takes part in the penetration of *E. coli* into brain microvascular endothelial cells. In the present study, the gene *ibeA* occurred only in 12.1% of *E. coli* lac+ strains and 8.6% of lac− strains, but none of the tested strains came from blood. In the earlier work, a significantly higher frequency of this gene was indicated among *E. coli* K1 (35.8%) than in strains without the capsule K1 (4.5%) (Kaczmarek et al. 2012).

*E. coli* rods may cause serious parenteral infections, and they are particularly dangerous for pregnant women and neonates. In the present study, it was indicated that there were more strains resistant to at least one antibiotic in the group of lac− isolates, and the frequency of genes encoding chosen virulence factors was similar in both groups of strains. *E. coli* lac+ and lac− strains had also common profiles of drug susceptibility and tested VGs. This may indicate their very similar pathogenic potential.

## Conclusion

It is essential to pay attention to the presence of lac− strains of *E. coli* in clinical material in the course of diagnostic procedures and to carry out their species identification. Omitting *E. coli* lac− strains as a potential etiological agent of infections may pose a threat to the health and life of both mothers and newborn babies.
